# Supplementary material for: A Comparison of Gene Expression Profiles of Rat Tissues after Mild and Short-Term Calorie Restrictions
Source: Nutrients. 2021 Jun 30;13(7):2277. doi: 10.3390/nu13072277 (PMC8308279; doi:10.3390/nu13072277)
Supplement: Supplementary file 1 [file nutrients-13-02277-s001.zip › Table S1.pdf]

**Supplementary Table 1** Number of genes altered by short-term and mild-to-moderate CR

| | | | 5% | 10% | 20% | 30% | Overlap† |
|---|---|---|---|---|---|---|---|
| liver | Up | 1 week | 69 | 420 | 613 | 326 | 40 |
| | | 1 month | 347 | 286 | 384 | 363 | |
| | Down | 1 week | 114 | 168 | 289 | 408 | 50 |
| | | 1 month | 148 | 168 | 188 | 207 | |
| | Up + Down | 1 week | 183 | 588 | 902 | 734 | 90 |
| | | 1 month | 495 | 454 | 572 | 570 | |

| | | | 5% | 10% | 20% | 30% | Overlap† |
|---|---|---|---|---|---|---|---|
| adipose | Up | 1 week | 247 | 585 | 653 | 915 | 45 |
| | | 1 month | 117 | 426 | 800 | 485 | |
| | Down | 1 week | 140 | 396 | 431 | 759 | 14 |
| | | 1 month | 187 | 806 | 824 | 770 | |
| | Up + Down | 1 week | 387 | 981 | 1084 | 1674 | 59 |
| | | 1 month | 304 | 1232 | 1624 | 1255 | |

| | | | 5% | 10% | 20% | 30% | Overlap† |
|---|---|---|---|---|---|---|---|
| muscle | Up | 1 week | 365 | 363 | 588 | 672 | 50 |
| | | 1 month | 496 | 565 | 525 | 690 | |
| | Down | 1 week | 319 | 300 | 641 | 845 | 51 |
| | | 1 month | 545 | 532 | 549 | 732 | |
| | Up + Down | 1 week | 684 | 663 | 1229 | 1517 | 101 |
| | | 1 month | 1041 | 1097 | 1074 | 1422 | |

| | | | 5% | 10% | 20% | 30% | Overlap† |
|---|---|---|---|---|---|---|---|
| brain (hypothalamus) | Up | 1 week | 350 | 417 | 433 | 349 | 0 |
| | | 1 month | 59 | 37 | 8 | 69 | |
| | Down | 1 week | 996 | 996 | 996 | 996 | 3 |
| | | 1 month | 17 | 21 | 111 | 79 | |
| | Up + Down | 1 week | 1346 | 1413 | 1429 | 1345 | 3 |
| | | 1 month | 76 | 58 | 119 | 148 | |

| | | | 5% | 10% | 20% | 30% | Overlap† |
|---|---|---|---|---|---|---|---|
| intestine | Up | 1 week | - | - | - | - | 47 |
| | | 1 month | 284 | 293 | 214 | 425 | |
| | Down | 1 week | - | - | - | - | 23 |
| | | 1 month | 86 | 210 | 316 | 133 | |
| | Up + Down | 1 week | - | - | - | - | 70 |
| | | 1 month | 370 | 503 | 530 | 558 | |

† The number of gene probes overlapped in the same direction throughout all the CR groups. The values were determined according to the criteria described in Materials and methods. 5%: 5% calorie restriction. 10%: 10% calorie restriction. 20%: 20% calorie restriction. 30%: 30% calorie restriction.
