# Supplementary material for: A Comparison of Gene Expression Profiles of Rat Tissues after Mild and Short-Term Calorie Restrictions
Source: Nutrients. 2021 Jun 30;13(7):2277. doi: 10.3390/nu13072277 (PMC8308279; doi:10.3390/nu13072277)
Supplement: Supplementary file 1 [file nutrients-13-02277-s001.zip › Table S2&S3.pdf]

**Supplementary Table 2** GO analysis of the genes up-regulated by 30%CR

| | GO ID | GO Term (BP_Direct) | p-value |
|---|---|---|---|
| liver | GO:0055114 | oxidation-reduction process | 8.65E-13 |
| | GO:0006635 | fatty acid beta-oxidation | 1.39E-06 |
| | GO:0017144 | drug metabolic process | 1.68E-04 |
| adipose | GO:0055114 | oxidation-reduction process | 1.08E-22 |
| | GO:0006099 | tricarboxylic acid cycle | 1.63E-13 |
| | GO:0006635 | fatty acid beta-oxidation | 1.10E-11 |
| | GO:0033539 | fatty acid beta-oxidation using acyl-CoA dehydrogenase | 1.11E-06 |
| | GO:0055088 | lipid homeostasis | 7.19E-05 |
| | GO:0006629 | lipid metabolic process | 1.21E-04 |
| | GO:0008152 | metabolic process | 2.72E-04 |
| muscle | not identified | | |
| brain (hypothalamus) | not identified | | |
| intestin | GO:0007568 | aging | |

Adjusted p- value < 1×10-4 (Fisher's exact test)

**Supplementary Table 3** GO analysis of the genes down-regulated by 30%CR

| | GO ID | GO Term (BP_Direct) | p-value |
|---|---|---|---|
| liver | GO:0006695 | cholesterol biosynthetic process | 8.90E-12 |
| | GO:0006629 | lipid metabolic process | 4.03E-05 |
| | GO:0042493 | response to drug | 8.05E-04 |
| | GO:0016126 | sterol biosynthetic process | 8.05E-04 |
| | GO:0055114 | oxidation-reduction process | 8.05E-04 |
| adipose | GO:0098609 | cell-cell adhesion | 2.49E-12 |
| | GO:0019886 | antigen processing and presentation of exogenous peptide antigen via MHC class II | 9.61E-05 |
| | GO:0001525 | angiogenesis | 9.61E-05 |
| | GO:0042493 | response to drug | 9.61E-05 |
| | GO:0016064 | immunoglobulin mediated immune response | 9.61E-05 |
| | GO:0071260 | cellular response to mechanical stimulus | 9.61E-05 |
| | GO:0043066 | negative regulation of apoptotic process | 9.61E-05 |
| | GO:0043123 | positive regulation of I-kappaB kinase/NF-kappaB signaling | 1.56E-04 |
| | GO:0030036 | actin cytoskeleton organization | 2.99E-04 |
| | GO:0071407 | cellular response to organic cyclic compound | 4.88E-04 |
| | GO:0008360 | regulation of cell shape | 6.83E-04 |
| | GO:0045087 | innate immune response | 8.53E-04 |
| muscle | GO:0001666 | response to hypoxia | 1.29E-04 |
| | GO:0006936 | muscle contraction | 3.50E-04 |
| | GO:0030036 | actin cytoskeleton organization | 5.90E-04 |
| brain (hypothalamus) | not identified | | |
| intestin | not identified | | |

Adjusted p- value < 1×10-4 (Fisher's exact test)
