# Supplementary material for: A Comparison of Gene Expression Profiles of Rat Tissues after Mild and Short-Term Calorie Restrictions
Source: Nutrients. 2021 Jun 30;13(7):2277. doi: 10.3390/nu13072277 (PMC8308279; doi:10.3390/nu13072277)
Supplement: Supplementary file 1 [file nutrients-13-02277-s001.zip › Table S4.pdf]

SupplementaryTable 4 Genes comonly altered by CR

Up-regulated across all tissues	# of overlapped gene probes	Down-regulated across all tissues	# of overlapped gene probes
nuclear receptor subfamily 1, group D, member 2	29	CKLF-like MARVEL transmembrane domain containing 6	25
aldo-keto reductase family 1, member C14	21	cytochrome P450, family 51	23
nicotinamide phosphoribosyltransferase	21	heat shock protein 90, alpha (cytosolic), class A member 1 /// heat shock protein HSP 90-alpha /// similar to heat shock protein 1, alpha	21
glutamate-ammonia ligase	20	actin, gamma 1 /// actin, gamma 1 propeptide-like /// POTE ankyrin domain family, member F	20
sulfotransferase family 1A member 1	20	fatty acid desaturase 1	19
glutamate-ammonia ligase	20	tubulin, beta 4B class IVb	19
CD36 molecule (thrombospondin receptor) /// scavenger receptor class B, member 2-like /// platelet glycoprotein 4-like	20	tropomyosin 4	19
flavin containing monooxygenase 1	20	sphingosine-1-phosphate receptor 1	19
epoxide hydrolase 1, microsomal (xenobiotic)	20	tubulin, beta 2A class IIa	19
D site of albumin promoter (albumin D-box) binding protein	20	regulator of calcineurin 1	19
nuclear receptor subfamily 1, group D, member 2	19	clock circadian regulator	19
retinol saturase (all trans retinol 13,14 reductase)	19	tubulin alpha-1C chain-like /// tubulin, alpha 1A /// tubulin, alpha 1B /// tubulin, alpha 1C	18
Sad1 and UNC84 domain containing 2	19	Fc fragment of IgG, low affinity IIa, receptor /// Low affinity immunoglobulin gamma Fc region receptor III-like /// low affinity immunoglobulin gamma Fc region receptor III-like /// low affinity immunoglobulin gamma Fc region receptor III /// low affinity immunoglobulin gamma Fc region receptor III-like /// Fc gamma receptor II beta	18
patatin-like phospholipase domain containing 7	19	7-dehydrocholesterol reductase	18
ubiquitin specific peptidase 2	19	transmembrane protein 97	18
xanthine dehydrogenase	18	early growth response 1	17
PTEN induced putative kinase 1	18	tubulin, alpha 4A	17
sphingomyelin synthase 1	18	serine/arginine-rich splicing factor 3	17
enoyl CoA hydratase 1, peroxisomal	18	glycine amidinotransferase (L-arginine:glycine amidinotransferase)	16
carnitine palmitoyltransferase 1a, liver	18	isopentenyl-diphosphate delta isomerase 1	16
P450 (cytochrome) oxidoreductase	18	cysteine and glycine-rich protein 1	16
BCL2/adenovirus E1B interacting protein 3	18	spermidine/spermine N1-acetyl transferase 1	16
glutamate-ammonia ligase	17	perilipin 2	16
CD36 molecule (thrombospondin receptor) /// scavenger receptor class B, member 2-like	17	proline rich 13	16
2,4-dienoyl CoA reductase 1, mitochondrial	17	transferrin receptor	16
Kruppel-like factor 15	17	nuclear receptor subfamily 4, group A, member 3	15
Kruppel-like factor 9	17	sterol regulatory element binding transcription factor 1	15
G protein-coupled receptor 146	17	low density lipoprotein receptor	15
homocysteine-inducible, endoplasmic reticulum stress-inducible, ubiquitin-like domain member 1	16	sterol regulatory element binding transcription factor 1	15
P450 (cytochrome) oxidoreductase	16	heat shock 105/110 protein 1	15
aldo-keto reductase family 1, member C14	16	transmembrane protein 140	15
diacylglycerol O-acyltransferase 2	16	Fc fragment of IgG, low affinity IIa, receptor /// low affinity immunoglobulin gamma Fc region receptor III-like /// low affinity immunoglobulin gamma Fc region receptor III /// low affinity immunoglobulin gamma Fc region receptor III-like /// Fc gamma receptor II beta	15
perilipin 4	16	glutaredoxin (thioltransferase)	14
eukaryotic translation initiation factor 4E binding protein 1	16	insulin induced gene 1	14
guanine deaminase	16	methylsterol monooxygenase 1	14
matrix Gla protein	15	dynein light chain LC8-type 1	14
aldehyde dehydrogenase 3 family, member A2	15	cofilin 1, non-muscle	14
methyltransferase like 7A	15	signal transducer and activator of transcription 3 (acute-phase response factor)	14
GABA(A) receptor-associated protein like 1	15	signal peptidase complex subunit 3	14
sideroflexin 1	15	heat shock protein 90, alpha (cytosolic), class A member 1 /// heat shock protein 1, alpha-like /// heat shock protein HSP 90-alpha	14
indolethylamine N-methyltransferase	15	interferon regulatory factor 2 binding protein 2	14
oxysterol binding protein-like 11	15	sigma non-opioid intracellular receptor 1	14
metallothionein 2A	15	BTG family, member 2	14
glycerophosphodiester phosphodiesterase 1	15	cytoplasmic polyadenylation element binding protein 2	14
glycogen synthase 2	14	matrix metallopeptidase 14 (membrane-inserted)	13
sorbitol dehydrogenase	14	calmodulin 1	13
hydroxyacyl-CoA dehydrogenase/3-ketoacyl-CoA thiolase/enoyl-CoA hydratase (trifunctional protein), alpha subunit	14	calmodulin 1	13

cytochrome P450, family 2, subfamily b, polypeptide 1 /// cytochrome P450, family 2, subfamily b, polypeptide 2	14	lysozyme 2	13
metallothionein 1a /// transthyretin	14	malic enzyme 1, NADP(+)-dependent, cytosolic	13
patatin-like phospholipase domain-containing protein 2-like /// patatin-like phospholipase domain containing 2	14	WD repeat and SOCS box-containing 1	13
tetraspanin-4-like /// tetraspanin 4	14	glutaredoxin (thioltransferase)	13
pleckstrin homology-like domain, family A, member 3	14	BTG family, member 2	13
sorbin and SH3 domain containing 1	14	UDP-Gal:betaGlcNAc beta 1,4-galactosyltransferase, polypeptide 6	13
cordon-bleu WH2 repeat protein-like 1	14	exportin 1	13
phytanoyl-CoA 2-hydroxylase	14	G0/G1switch 2	13
phosphoinositide-3-kinase interacting protein 1	14	dual-specificity tyrosine-(Y)-phosphorylation regulated kinase 2	13
FK506 binding protein 5	14	actin, beta	13
aconitase 2, mitochondrial	13	B-cell translocation gene 1, anti-proliferative	12
enoyl-CoA delta isomerase 1	13	farnesyl diphosphate synthase	12
microtubule-associated protein 1 light chain 3 beta	13	fatty acid synthase	12
selenium-binding protein 1 /// selenium binding protein 1	13	clusterin	12
glutathione S-transferase alpha 1 /// glutathione S-transferase alpha-3-like	13	HOP homeobox	12
nuclear protein, transcriptional regulator, 1 /// nuclear protein, transcriptional regulator, 1	13	malic enzyme 1, NADP(+)-dependent, cytosolic	12
mitochondrial amidoxime reducing component 2	13	collagen, type I, alpha 2 /// collagen, type I, alpha 2	12
enoyl-CoA, hydratase/3-hydroxyacyl CoA dehydrogenase	13	transformer 2 beta homolog (Drosophila)	12
pyruvate dehydrogenase kinase, isozyme 4	13	heat shock protein 5	12
protein tyrosine phosphatase 4a1-like /// protein tyrosine phosphatase type IVA 1 /// protein tyrosine phosphatase type IVA, member 1	13	proline-rich nuclear receptor coactivator 1	12
solute carrier family 38, member 3	13	ARP3 actin-related protein 3 homolog (yeast)	12
starch binding domain 1	13	related RAS viral (r-ras) oncogene homolog 2	12
yippee-like 3	13	glycerol-3-phosphate acyltransferase, mitochondrial	12
vascular endothelial growth factor A	13	vasodilator-stimulated phosphoprotein	12
arrestin domain containing 3	13	myristoylated alanine rich protein kinase C substrate	12
myosin regulatory light chain interacting protein	13	Tnf receptor-associated factor 3	12
uncharacterized LOC100910069 /// uncharacterized LOC102554001 /// similar to NS1-associated protein 1 isoform 2	13	phosphodiesterase 4B, cAMP specific	12
hydroxyacyl-CoA dehydrogenase/3-ketoacyl-CoA thiolase/enoyl-CoA hydratase (trifunctional protein), beta subunit	12	myristoylated alanine rich protein kinase C substrate	12
Pim-3 proto-oncogene, serine/threonine kinase	12	actin related protein 2/3 complex, subunit 5-like	12
carnitine palmitoyltransferase 1a, liver	12	tetratricopeptide repeat domain 7A	12
flavin containing monooxygenase 3	12	cysteine-rich with EGF-like domains 2	12
basic helix-loop-helix family, member e41	12	CD164 molecule, sialomucin	12
sorbitol dehydrogenase	12	carbonic anhydrase 2	12
glutamate cysteine ligase, modifier subunit	12	solute carrier family 16 (monocarboxylate transporter), member 1	12
2-hydroxyacyl-CoA lyase 1	12	cytochrome P450, family 51	12
ankyrin repeat and KH domain containing 1 /// eukaryotic translation initiation factor 4E binding protein 3	12	transmembrane protein 263	12
hydroxysteroid (17-beta) dehydrogenase 11	12	COMM domain containing 6	12
cell growth regulator with ring finger domain 1	12	transmembrane protein 100	12
UDP-glucose pyrophosphorylase 2	12	perilipin 2	12
complement factor D (adipsin)	12	CD44 molecule (Indian blood group)	12
zinc finger protein 709-like	12	lactate dehydrogenase A	11
sulfatase 2	12	stearoyl-Coenzyme A desaturase 2	11
Rho-related BTB domain containing 1	12	high mobility group box 2 /// high mobility group box 2-like 1 /// similar to High mobility group protein 2 (HMG-2)	11
uracil-DNA glycosylase	12	ATP citrate lyase	11
Cbp/p300-interacting transactivator, with Glu/Asp-rich carboxy-terminal domain, 2	11	adenosylmethionine decarboxylase 1	11
alpha-methylacyl-CoA racemase	11	Kirsten rat sarcoma viral oncogene	11
nuclear factor, erythroid 2-like 2	11	ornithine decarboxylase 1	11
ribonuclease, RNase A family 4	11	tropomyosin 3	11
6-phosphofructo-2-kinase/fructose-2,6-biphosphatase 1	11	myristoylated alanine rich protein kinase C substrate	11
solute carrier family 25 (mitochondrial carrier; dicarboxylate transporter), member 10	11	serpin peptidase inhibitor, clade H (heat shock protein 47), member 1, (collagen binding protein 1)	11
cytochrome P450, family 4, subfamily b, polypeptide 1	11	adiponectin receptor 2	11
spectrin, beta, non-erythrocytic 1	11	serine/arginine-rich splicing factor 2	11
RNA binding motif (RNP1, RRM) protein 3	11	tripartite motif-containing 5	11
similar to RIKEN cDNA 4931406C07	11	protein kinase domain containing, cytoplasmic	11
methionine sulfoxide reductase B1	11	immunoglobulin heavy chain, alpha /// similar to immunoglobulin heavy chain	11
gap junction protein, alpha 1	11	similar to hypothetical protein A530094D01	11
patatin-like phospholipase domain containing 8	11	heat shock protein 90 alpha (cytosolic), class B member 1	11

procollagen C-endopeptidase enhancer 2	11	v-maf avian musculoaponeurotic fibrosarcoma oncogene homolog	11
mitochondrial amidoxime reducing component 1	11	transketolase	11
dual specificity phosphatase 6	11	tubulin, beta 5 class I	11
angiotensin II receptor, type 1a	11	phosphatidylinositol-5-phosphate 4-kinase, type II, alpha	11
carnitine palmitoyltransferase 2	11	FK506 binding protein 4	11
acyl-CoA synthetase long-chain family member 4	11	bone marrow stromal cell antigen 2	11
osteomodulin /// osteomodulin	11	akirin 1	11
acetyl-CoA acyltransferase 1A /// acetyl-Coenzyme A acyltransferase 1B	11	DnaJ (Hsp40) homolog, subfamily A, member 1	11
Fc fragment of IgG, receptor, transporter, alpha	11	core-binding factor, beta subunit	11
prostaglandin reductase 1	11	profilin 1	10
zinc binding alcohol dehydrogenase, domain containing 2	11	syndecan 4	10
solute carrier family 25 (carnitine/acylcarnitine translocase), member 20	11	carbonic anhydrase 2	10
malonyl-CoA decarboxylase	10	ADP-ribosylation factor 5	10
receptor (G protein-coupled) activity modifying protein 2	10	interferon-related developmental regulator 1	10
cytochrome P450, family 2, subfamily e, polypeptide 1	10	3-hydroxy-3-methylglutaryl-CoA synthase 1 (soluble)	10
adenylate kinase 3	10	CAP, adenylate cyclase-associated protein 1 (yeast)	10
ectonucleotide pyrophosphatase/phosphodiesterase 2	10	caldesmon 1	10
phosphodiesterase 3B, cGMP-inhibited	10	tropomyosin 4	10
coagulation factor III (thromboplastin, tissue factor)	10	transmembrane protein 176B	10
isocitrate dehydrogenase 1 (NADP+), soluble	10	argonaute RISC catalytic component 2	10
lactate dehydrogenase B	10	inhibitor of DNA binding 2	10
peripheral myelin protein 22	10	cAMP responsive element modulator	10
3-hydroxy-3-methylglutaryl-CoA synthase 2 (mitochondrial)	10	fatty acid binding protein 7, brain	10
nudix (nucleoside diphosphate linked moiety X)-type motif 6	10	proteasome subunit beta 9	10
nuclear receptor subfamily 1, group D, member 2	10	fatty acid binding protein 5, epidermal	10
nuclear receptor subfamily 1, group D, member 1	10	insulin-like growth factor binding protein 5	10
glycerol-3-phosphate dehydrogenase 1 (soluble)	10	TAR DNA binding protein	10
brain protein I3	10	24-dehydrocholesterol reductase	10
monocyte to macrophage differentiation-associated	10	ubiquitin-conjugating enzyme E2H	10
rhomboid domain containing 2	10	ornithine decarboxylase antizyme 1	10
MID1 interacting protein 1	10	ST3 beta-galactoside alpha-2,3-sialyltransferase 1	10
solute carrier family 4, sodium bicarbonate cotransporter, member 4	10	SKI-like proto-oncogene	10
aarF domain containing kinase 3	10	uncharacterized LOC100910973	10
ilvB (bacterial acetolactate synthase)-like	10	oligosaccharyltransferase complex subunit (non-catalytic)	10
carbonic anhydrase 14	10	sorcin	10
interleukin 33	10	family with sequence similarity 213, member A	10
family with sequence similarity 214, member A	10	M phase phosphoprotein 6	10
asporin /// asporin	10	Kruppel-like factor 2	10
acetyl-CoA acyltransferase 2	10	mesenteric estrogen-dependent adipogenesis	10
solute carrier family 9, subfamily A (NHE3, cation proton antiporter 3), member 3 regulator 1	10	interferon, alpha-inducible protein 27 like 2B	10
t-complex 11, testis-specific-like 2	10	dual-specificity tyrosine-(Y)-phosphorylation regulated kinase 2	10
solute carrier family 25, member 42	10	cAMP responsive element modulator	10
schlafen family member 5	10	related RAS viral (r-ras) oncogene homolog 2	10
nudix (nucleoside diphosphate linked moiety X)-type motif 7	10	APEX nuclease (multifunctional DNA repair enzyme) 1	10
meteorin, glial cell differentiation regulator-like	10	glucose-6-phosphatase, catalytic subunit	10
spectrin, beta, non-erythrocytic 1	10	SH3-domain binding protein 5 (BTK-associated)	10
transmembrane protein 57	10	progesterone receptor membrane component 2	10
CDC-like kinase 1	10	hematological and neurological expressed 1	10
		chloride intracellular channel 4	10

Up-regulated in liver	# of overlapped gene probes	Down-regulated in liver	# of overlapped gene probes
enoyl-CoA delta isomerase 1	8	tubulin alpha-1C chain-like /// tubulin, alpha 1A /// tubulin, alpha 1B /// tubulin, alpha 1C	8
carnitine palmitoyltransferase 1a, liver	8	farnesyl diphosphate synthase	8
P450 (cytochrome) oxidoreductase	8	protein kinase C, delta binding protein	8
cytochrome P450, family 7, subfamily a, polypeptide 1	8	fatty acid synthase	8
ectonucleotide pyrophosphatase/phosphodiesterase 2	8	fatty acid synthase	8
nuclear receptor subfamily 1, group I, member 3	8	ATP citrate lyase	8
acyl-CoA thioesterase 12	8	insulin induced gene 1	8
integral membrane transport protein UST4r	8	cytochrome P450, family 51	8
serine dehydratase	8	7-dehydrocholesterol reductase	8
cytochrome P450, family 4, subfamily a, polypeptide 3	8	aquaporin 8	8
2-hydroxyacyl-CoA lyase 1	8	pyruvate kinase, liver and RBC	8
metallothionein 1a /// transthyretin	8	catechol-O-methyltransferase	8
ankyrin repeat domain 46	8	arginine vasopressin receptor 1A	8

rhomboid domain containing 2	8	malic enzyme 1, NADP(+)-dependent, cytosolic	8
F-box protein 31	8	fatty acid binding protein 5, epidermal	8
sideroflexin 1	8	epidermal growth factor receptor	8
ankyrin repeat and KH domain containing 1 /// eukaryotic translation initiation factor 4E binding protein 3	8	malic enzyme 1, NADP(+)-dependent, cytosolic	8
indolethylamine N-methyltransferase	8	lipopolysaccharide-induced TNF factor	8
folliculin interacting protein 2	8	sterol regulatory element binding transcription factor 1	8
phosphatase domain containing, paladin 1	8	actin, gamma 1 /// actin, gamma 1 propeptide-like /// POTE ankyrin domain family, member F	8
cordon-bleu WH2 repeat protein-like 1	8	tubulin, beta 4B class IVb	8
enoyl CoA hydratase 1, peroxisomal	8	tubulin, alpha 4A	8
carnitine palmitoyltransferase 2	8	adiponectin receptor 2	8
carnitine palmitoyltransferase 1a, liver	8	phosphogluconate dehydrogenase	8
aldehyde dehydrogenase 1 family, member A1	8	spermidine/spermine N1-acetyl transferase 1	8
flavin containing monooxygenase 1	8	CKLF-like MARVEL transmembrane domain containing 6	8
P450 (cytochrome) oxidoreductase	8	transmembrane protein 97	8
hydroxysteroid (17-beta) dehydrogenase 2	8	MARVEL domain containing 1	8
N-acetyltransferase 8	8	glycerol-3-phosphate acyltransferase, mitochondrial	8
calponin 3, acidic	8	phosphomevalonate kinase	8
metallothionein 2A	8	paroxysmal nonkinesigenic dyskinesia	8
zinc finger protein 709-like	8	tubulin, beta 2A class IIa	8
protein phosphatase, Mg2+/Mn2+ dependent, 1K	8	selenocysteine lyase	8
uracil-DNA glycosylase	8	pyroglutamyl-peptidase I	8
nuclear receptor subfamily 1, group D, member 2	8	claudin 2	8
solute carrier family 16, member 12	8	acyl-CoA synthetase short-chain family member 2	8
glycerophosphodiester phosphodiesterase 1	8	caseinolytic mitochondrial matrix peptidase chaperone subunit	8
		YEATS domain containing 4	8
		sigma non-opioid intracellular receptor 1	8
		solute carrier family 16 (monocarboxylate transporter), member 1	8
		glucokinase	8
		sterol regulatory element binding transcription factor 1	8
		COMM domain containing 6	8
		insulin induced gene 2	8
		transmembrane protein 140	8
		perilipin 2	8

Up-regulated in apipose	# of overlapped gene probes	Down-regulated in adipose	# of overlapped gene probes
glutamate-ammonia ligase	8	clusterin	8
carnitine palmitoyltransferase 1b, muscle	8	secretory leukocyte peptidase inhibitor	8
diacylglycerol O-acyltransferase 1	8	7-dehydrocholesterol reductase	8
carbonyl reductase 1 /// inducible carbonyl reductase-like /// carbonyl reductase [NADPH] 1-like	8	deoxyribonuclease 1-like 3	8
P450 (cytochrome) oxidoreductase	8	lysozyme 2	8
steroidogenic acute regulatory protein	8	AHA1, activator of heat shock 90kDa protein ATPase homolog 1 (yeast)	8
phosphodiesterase 3B, cGMP-inhibited	8	tubulin, beta 2A class IIa	8
coagulation factor III (thromboplastin, tissue factor)	8	milk fat globule-EGF factor 8 protein	8
melanoma cell adhesion molecule	8	ficolin A	8
isocitrate dehydrogenase 1 (NADP+), soluble	8	transferrin receptor	8
optineurin	8	heat shock 105/110 protein 1	8
protein kinase, cAMP dependent regulatory, type II beta	8	macrophage expressed 1	8
monocyte to macrophage differentiation-associated	8	patatin-like phospholipase domain containing 3	8
paternally expressed 3	8	Fc fragment of IgG, low affinity IIa, receptor /// low affinity immunoglobulin gamma Fc region receptor III-like /// low affinity immunoglobulin gamma Fc region receptor III /// low affinity immunoglobulin gamma Fc region receptor III-like /// Fc gamma receptor II beta	8
aarF domain containing kinase 3	8		
tetraspanin-4-like /// tetraspanin 4	8		
ilvB (bacterial acetolactate synthase)-like	8		
transmembrane protein 120A	8		
CTD (carboxy-terminal domain, RNA polymerase II, polypeptide A) small phosphatase-like	8		
1-acylglycerol-3-phosphate O-acyltransferase 2	8		
retinol binding protein 7, cellular	8		
asparaginase like 1	8		
melanoma cell adhesion molecule	8		
sorbin and SH3 domain containing 1	8		

peroxisomal biogenesis factor 1	8
salt-inducible kinase 2	8
mitochondrial amidoxime reducing component 1	8
mitochondrial amidoxime reducing component 1	8
glutamate-ammonia ligase	8
regulator of G-protein signaling 2	8
P450 (cytochrome) oxidoreductase	8
lipase, hormone sensitive	8
steroidogenic acute regulatory protein	8
potassium channel, two pore domain subfamily K, member 3	8
programmed cell death 4	8
epoxide hydrolase 1, microsomal (xenobiotic)	8
BCL2/adenovirus E1B interacting protein 3	8
D site of albumin promoter (albumin D-box) binding protein	8
asparaginase like 1	8
gamma-aminobutyric acid (GABA) B receptor 1	8
acyl-CoA synthetase long-chain family member 1	8
optineurin	8
nicotinamide phosphoribosyltransferase	8
CTP synthase 2	8
potassium channel, two pore domain subfamily K, member 3	8

Up-regulated in muscle	# of overlapped gene probes	Down-regulated in muscle	# of overlapped gene probes
CD36 molecule (thrombospondin receptor) /// scavenger receptor class B, member 2-like	8	glycine amidinotransferase (L-arginine:glycine amidinotransferase)	8
nuclear factor, erythroid 2-like 2	8	HOP homeobox	8
interleukin 15	8	fatty acid desaturase 1	8
synaptojanin 2	8	nuclear factor, interleukin 3 regulated	8
pyruvate dehydrogenase kinase, isozyme 4	8	myosin binding protein H	8
thyrotrophic embryonic factor	8	phosphodiesterase 4B, cAMP specific	8
xanthine dehydrogenase	8	insulin-like growth factor binding protein 5	8
sulfotransferase family 1A member 1	8	tumor necrosis factor receptor superfamily, member 12a	8
nuclear receptor subfamily 1, group D, member 2	8	CKLF-like MARVEL transmembrane domain containing 6	8
solute carrier family 38, member 3	8	related RAS viral (r-ras) oncogene homolog 2	8
spectrin, beta, non-erythrocytic 1	8	ST3 beta-galactoside alpha-2,3-sialyltransferase 1	8
diacylglycerol O-acyltransferase 2	8	anoctamin 6	8
MID1 interacting protein 1	8	actin filament associated protein 1-like 1	8
four and a half LIM domains 3	8	G protein-coupled receptor, class C, group 5, member C	8
zinc finger protein 703	8	WD repeat and SOCS box-containing 1	8
pericentrin	8	sideroflexin 5	8
sphingomyelin synthase 1	8	tight junction associated protein 1	8
pleckstrin homology-like domain, family A, member 3	8	mesenteric estrogen-dependent adipogenesis	8
ubiquitin specific peptidase 2	8	popeye domain containing 2	8
ubiquitin specific peptidase 2	8	ring finger protein 2	8
cysteine rich transmembrane BMP regulator 1 (chordin like)	8	dual-specificity tyrosine-(Y)-phosphorylation regulated kinase 2	8
sphingomyelin synthase 1	8	tetratricopeptide repeat domain 7A	8
scavenger receptor class A, member 5	8	nicotinamide riboside kinase 2	8
inositol polyphosphate-4-phosphatase, type II	8	cytokine inducible SH2-containing protein	8
ADP-ribosyltransferase 1	8	cAMP responsive element modulator	8
zinc finger protein 503	8	autism susceptibility candidate 2-like	8
CD36 molecule (thrombospondin receptor) /// scavenger receptor class B, member 2-like /// platelet glycoprotein 4-like	8	solute carrier family 16, member 9	8
carnitine palmitoyltransferase 1a, liver	8	protein tyrosine phosphatase, non-receptor type 4	8
flavin containing monooxygenase 1	8	ring finger protein 2	8
guanine deaminase	8	v-maf avian musculoaponeurotic fibrosarcoma oncogene homolog	8
epoxide hydrolase 1, microsomal (xenobiotic)	8	dual-specificity tyrosine-(Y)-phosphorylation regulated kinase 2	8
ubiquitin specific peptidase 2	8	dual-specificity tyrosine-(Y)-phosphorylation regulated kinase 2	8
D site of albumin promoter (albumin D-box) binding protein	8	v-maf avian musculoaponeurotic fibrosarcoma oncogene homolog	8
phosphoinositide-3-kinase interacting protein 1	8	SH3-domain binding protein 5 (BTK-associated)	8
actin-binding LIM protein 1	8	G0/G1switch 2	8
solute carrier family 25, member 42	8	heat shock protein 90, alpha (cytosolic), class A member 1 /// heat shock protein HSP 90-alpha /// similar to heat shock protein 1, alpha	8
nudix (nucleoside diphosphate linked moiety X)-type motif 7	8	PX domain containing 1	8
nuclear receptor subfamily 1, group D, member 2	8	clock circadian regulator	8
Rho GTPase activating protein 12	8	transmembrane protein 100	8
cysteine rich transmembrane BMP regulator 1 (chordin like)	8	PWWP domain containing 2B	8
zinc finger protein 503	8	dual-specificity tyrosine-(Y)-phosphorylation regulated kinase 2	8
peroxisome proliferator-activated receptor gamma, coactivator 1 alpha	8	clock circadian regulator	8

nicotinamide phosphoribosyltransferase	8	TEA domain family member 4	8
adhesion G protein-coupled receptor F5	8	similar to RIKEN cDNA D930015E06	8
cysteine rich transmembrane BMP regulator 1 (chordin like)	8	immunity-related GTPase family, M	8
		melanoma associated antigen (mutated) 1-like 1	8
		akirin 1	8
		solute carrier family 41, member 3	8
		uncharacterized LOC102554645 /// uncharacterized LOC103693891	8
		protein phosphatase 2, regulatory subunit B', alpha	8

Up-regulated in brain(hypothalamus)	# of overlapped gene probes	Down-regulated in brain(hypothalamus)	# of overlapped gene probes
		early growth response 1	8
		nuclear receptor subfamily 4, group A, member 3	8

Up-regulated in intestine	# of overlapped gene probes	Down-regulated in intestine	# of overlapped gene probes
matrix Gla protein	4	carbonic anhydrase 2	4
CD36 molecule (thrombospondin receptor) /// scavenger receptor class B, member 2-like	4	ADP-ribosylation factor 5	4
acyl-CoA dehydrogenase, C-4 to C-12 straight chain	4	glutaminase	4
glutathione S-transferase alpha 1 /// glutathione S-transferase alpha-3-like	4	abl-interactor 1	4
alpha-methylacyl-CoA racemase	4	heat shock 70kD protein 1A /// heat shock 70kD protein 1B (mapped)	4
2,4-dienoyl CoA reductase 1, mitochondrial	4	S100 calcium binding protein G	4
serum/glucocorticoid regulated kinase 1	4	regulator of G-protein signaling 4	4
claudin 3	4	neuromedin U	4
glycoprotein (transmembrane) nmb	4	signal transducer and activator of transcription 3 (acute-phase response factor)	4
dipeptidase 1 (renal)	4	Z-DNA binding protein 1	4
period circadian clock 2	4	2'-5' oligoadenylate synthetase 1A	4
carbonic anhydrase 4	4	chloride channel accessory 4	4
selenoprotein P, plasma, 1	4	carbonic anhydrase 2	4
fatty acid binding protein 1, liver	4	glucose-6-phosphatase, catalytic subunit	4
fatty acid binding protein 2, intestinal	4	lipoprotein lipase	4
solute carrier family 15 (oligopeptide transporter), member 1	4	BTG family, member 2	4
ATP-binding cassette, subfamily G (WHITE), member 5	4	alkaline phosphatase 3, intestine, not Mn requiring	4
solute carrier family 6 (proline IMINO transporter), member 20	4	heat shock 105/110 protein 1	4
sulfotransferase family 1A member 1	4	vanin 1	4
angiotensin I converting enzyme /// angiotensin-converting enzyme-like	4	palladin	4
peripheral myelin protein 22	4	serine/arginine repetitive matrix 2	4
cytochrome P450, family 3, subfamily a, polypeptide 9	4	DnaJ (Hsp40) homolog, subfamily A, member 1	4
cytochrome P450, family 4, subfamily b, polypeptide 1	4		
cytochrome P450, family 2, subfamily b, polypeptide 1 /// cytochrome P450, family 2, subfamily b, polypeptide 2	4		
metallothionein 1a /// transthyretin	4		
ADP-ribosyltransferase 3	4		
CNDP dipeptidase 2 (metallopeptidase M20 family)	4		
phosphoenolpyruvate carboxykinase 1 (soluble)	4		
glutamate-cysteine ligase, catalytic subunit	4		
ankyrin repeat and KH domain containing 1 /// eukaryotic translation initiation factor 4E binding protein 3	4		
Nedd4 family interacting protein 2	4		
CLOCK-interacting pacemaker	4		
hydroxyprostaglandin dehydrogenase 15 (NAD)	4		
lectin, galactoside-binding, soluble, 3	4		
CD36 molecule (thrombospondin receptor) /// scavenger receptor class B, member 2-like /// platelet glycoprotein 4-like	4		
histamine N-methyltransferase	4		
epoxide hydrolase 1, microsomal (xenobiotic)	4		
ubiquitin specific peptidase 2	4		
solute carrier family 9, subfamily A (NHE3, cation proton antiporter 3), member 3 regulator 1	4		
D site of albumin promoter (albumin D-box) binding protein	4		
mucin 3, intestinal	4		
metallothionein 2A	4		
chemokine (C-C motif) ligand 6	4		

myosin regulatory light chain interacting protein	4
transmembrane protein 45b	4
nuclear receptor subfamily 1, group D, member 2	4
carnosine dipeptidase 1 (metallopeptidase M20 family)	4
