# Supplementary material for: A Comparison of Gene Expression Profiles of Rat Tissues after Mild and Short-Term Calorie Restrictions
Source: Nutrients. 2021 Jun 30;13(7):2277. doi: 10.3390/nu13072277 (PMC8308279; doi:10.3390/nu13072277)
Supplement: Supplementary file 1 [file nutrients-13-02277-s001.zip › Table S5.pdf]

Supplementary Table 5 Reported meta-analysis of CR transcriptome experiment

	Ref #1	Ref #2
Reference	William R Swindell<br>BMC Genomics. 2009	Michael Plank et. al.<br>Mol Biosyst. 2012
# of studies	33	61
Organisms	mouse	mouse, rat, pig
Organs and tissues	Liver, Heart, Muscle, White Adipose Tissue, Hippocampus, Cortex, Hypothalamus, Cerebellum, Kidney, Lung, Thymus, Spinal Cord, Striatum, Cochlea, Gonads, Mammary Gland, Colon	Liver, Skeletal Muscle, Cochlea, Thymus, Kidney, Heart, Mammary Gland, Testis, Ovary, Lung, Adipose tissue, Neocortex, Forebrain, Gastrocnemius, Cerebellum, Hippocampus, SpinalCord, Striatum, Cortex, Colon, Hypothalamus
CR level	26 - 66%	between 10 and 40% (in most cases)
CR duration	1 - 31 month	within one day - 23.5 month
