# Supplementary material for: A Comparison of Gene Expression Profiles of Rat Tissues after Mild and Short-Term Calorie Restrictions
Source: Nutrients. 2021 Jun 30;13(7):2277. doi: 10.3390/nu13072277 (PMC8308279; doi:10.3390/nu13072277)
Supplement: Supplementary file 1 [file nutrients-13-02277-s001.zip › Table S6.pdf]

Supplementary Table 6 The comparison of the top five genes consistently up- or down- regulated for each organ with meta-analysis data

	Gene Symbol	Our Study	Ref #1	Ref #2
liver	Mt2A	up		up
	Mt1a /// Ttr	up		up
	Nat8	up	up	
	Hsd17b2	up	up	
	Por	up	up	up
adipose	Tubb2a	down		down
	Me1	down		down
	Acly	down	down	
	Dbp	up	up	down
	Dhcr7	down	down	
muscle	Fmo1	up		up
	Sult1a1	up	up	up
	G0s2	down	down	
brain	Egr1	down		down
intestin	Sult1a1	up	up	up
	Per2	up	up	up
	Abcg5	up	up	
	Hspa1a /// Hspa1b	down		down
